# Heterologous expression and biochemical characterization of a highly active and stable chloroplastic CuZn-superoxide dismutase from *Pisum sativum*

**DOI:** 10.1186/s12896-015-0117-0

**Published:** 2015-02-08

**Authors:** Narendra Tuteja, Panchanand Mishra, Sandep Yadav, Marjan Tajrishi, Sudhir Baral, Surendra Chandra Sabat

**Affiliations:** Plant Molecular Biology Group, International Centre for Genetic Engineering and Biotechnology (ICGEB), Aruna Asaf Ali Marg, New Delhi, 110067 India; Stress Biology Laboratory, Gene Function and Regulation, Institute of Life Sciences, Bhubaneswar, 751023 Odisha India

**Keywords:** Heterologous expression, CD spectroscopy, Homology modelling, Ni-NTA purification, Peroxidase activity, Superoxide dismutase

## Abstract

**Background:**

CuZn-Superoxide dismutase (SOD) is a unique enzyme, which can catalyzes the dismutation of inevitable metabolic product i.e.; superoxide anion into molecular oxygen and hydrogen peroxide. The enzyme has gained wide interest in pharmaceutical industries due to its highly acclaimed antioxidative properties. The recombinant expression of this protein in its enzymatically active and stable form is highly desired and hence optimization of culture conditions and characterization of the related biochemical properties are essential to explore the significance of the enzyme in physiological, therapeutic, structural and transgenic research.

**Results:**

High-level expression of the chloroplastic isoform of *Pisum sativum* CuZn-SOD was achieved at 18°C, upon isopropyl β-D-1-thiogalactopyranoside induction and the process was optimized for maximum recovery of the protein in its soluble (enzymatically active) form. Both crude and purified protein fractions display significant increase in activity following supplementation of defined concentration Cu (CuSO_4_) and Zn (ZnSO_4_). Yield of the purified recombinant protein was ~ 4 mg L^−1^ of culture volume and the bacterial biomass was ~ 4.5 g L^−1^. The recombinant pea chloroplastic SOD was found to possess nearly 6 fold higher superoxide dismutase activity and the peroxidase activity was also 5 fold higher as compared to commercially available CuZn-superoxide dismutase. The computational, spectroscopic and biochemical characterization reveals that the protein harbors all the characteristics features of this class of enzyme. The enzyme was found to be exceptionally stable as evident from pH and temperature incubation studies and maintenance of SOD activity upon prolonged storage.

**Conclusions:**

Overexpression and purification strategy presented here describes an efficient protocol for the production of a highly active and stable CuZn-superoxide dismutase in its recombinant form in *E. coli* system. The strategy can be utilized for the large-scale preparation of active CuZn-superoxide dismutase and thus it has wide application in pharmaceutical industries and also for elucidating the potential of this protein endowed with exceptional stability and activity.

**Electronic supplementary material:**

The online version of this article (doi:10.1186/s12896-015-0117-0) contains supplementary material, which is available to authorized users.

## Background

Superoxide dismutases (SOD, EC 1.15.1.1) are ubiquitous enzyme present in plants, animals and microbes and acts as the first line of defense against the toxic effects of superoxide (O_2_^∙−^) [1].These metallo-enzymes catalyze the dismutation of O_2_^∙−^ radicals to yield molecular oxygen (O_2_) and hydrogen peroxide (H_2_O_2_) [1,2]. Based on the metal ion cofactor identified in their active site, three types of SODs (CuZn-SOD, Fe-SOD and Mn-SOD) are reported to be present in the plant cell. Amongst these, CuZn-SODs are the most prominent form, and its isoforms have been reported to be present in cytosol, chloroplast, peroxisomes, and in extracellular location also [3,4]. In chloroplast, the CuZn -SOD serves as an important component of water–water cycle and hence prevents plant from photo-oxidative injury [5].

The chloroplastic CuZn-SOD display significant differences with cytosolic SODs in terms of their amino acid composition, spectral characteristics, sensitivity towards H_2_O_2_, and immunological properties [6]. The two isozymes are believed to evolved independently and diverged at very early stage of its acquisition [7]. In plant species like *Arabidopsis thaliana*, mustard, bean and citrus Fe-SOD is a major SOD isoform in the leaf, indicating that Fe-SOD alone could remove superoxide from photosynthesis process. Yet, in pea, the level of Fe-SOD is much lower as compared to chloroplastic CuZn-SOD, which could indicate that in these group of plants, chloroplastic CuZn-SOD is primarily involved in protecting chloroplasts from O_2_^.-^ mediated damage of photosynthetic machinery [8].

Except few cases, eukaryotic CuZn-SODs are generally homodimeric, consisting of two identical subunits with one copper (Cu^2+^) and one zinc (Zn^2+^) ion per subunit [9]. The two subunits are held together by hydrophobic interaction and the arrangement of amino acids is conserved at the dimer interface [10]. The three dimensional structure is characterized by a *β*-barrel structure composed of eight *β*-sheets with three loops, which are connected through disulfide bridge. The content of α-helical structure accounts for 5% only, exclusive to loop region. The active copper ion is solvent exposed and serves as redox partner for O_2_^∙−^, whereas the zinc ion is buried inside the *β*-barrel and is crucial for stabilization of its structure [11]. CuZn-SODs are believed to be one of the most stable globular protein families studied so far. They retain the native structure in 8 M urea, 4% SDS, has a melting temperature as high as 80°C and are also resistant to proteolytic degradation [12]. Several factors contribute to the enzyme’s exceptional stability, including the close packing of hydrophobic interfaces in the dimeric eukaryotic enzymes, the tight packing of the eight stranded *β* -barrel scaffold, the presence of an intra-chain disulfide bridge and the stabilizing effect of metal cofactors [13,14]. In spite of the stringent conservation of the three dimensional structure displayed by all the eukaryotic CuZn-SODs, enzymes isolated from various natural sources often exhibit difference in their kinetic properties and stability [12]. The study of the molecular determinants that are responsible for those differences could provide hints to understand factors governing protein stability and activity.

The heterologous expression of CuZn-SODs in bacteria and yeast often results in the impairment of recombinant protein activity [15]. This may be due to formation of apo-protein and/or improper folding of the protein in its recombinant form. Nevertheless, the overexpression studies are usually accompanied by supplementation of copper and zinc in growth medium [16,17]. In yeast, copper regulates the expression of cytosolic CuZn-SOD at both transcriptional and post-translational levels, while there is no copper-mediated transcriptional regulation of CuZn-SOD in human cells and higher plants, instead there is a translational and/or post-translational mechanism for CuZn-SOD activation [18].

SOD is a unique enzyme which can eliminate O_2_^∙−^ and therefore the enzyme is widely used in parenteral administrative in case of various diseases principally related to oxidative stress like musculoskeletal inflammation, Alzheimer disease, Crohon’s disease, intestinal fat absorption, and as an anti-inflammatory agent [19]. It is also used as an oral administration for radiation therapy, treating ulcers in the eye cornea and for reducing aging symptoms such as wrinkles etc. [19]. Thus, importance of SOD has been acknowledged worldwide in pharmaceutical industries and there are various efforts to develop more efficient and stable enzyme and improvement of *in vivo* pharmacological activity of SOD by conjugating with polysaccharides, hemoglobin and lecithin [20,21].

In the present study, we describe the recombinant expression and biochemical characterization of a highly active chloroplastic CuZn-SOD from *Pisum sativum* (PschSOD) in *E. coli* expression system. The bacterial growth conditions were optimized for the recovery of maximum protein in the enzymatically active (soluble) form. The recombinantly purified protein was further examined for its spectral and biochemical characteristics. The protein displays very high activity (both O_2_^∙−^ dismutase and peroxidase), and remains active under a broad range of pHs. The enzyme is stable to proteolytic digestion and 50% of its original activity is retained even after storing for 180 days at room temperature (25°C). Owing to the properties mentioned above, the recombinant PschSOD may find its utility in the medical, cosmetics, and food industries. It may also be valuable for producing transgenic plants that need additional tolerance to biotic and abiotic stresses [22]. Thus, the present study provides an excellent strategy to produce recombinant CuZn-SOD in its highly active and stable form.

## Results and discussion

### PCR amplification and cloning of PschSOD

To obtain the sequence of PschSOD without ‘Chloroplast Transit Peptide’, internal primers were synthesized and specific product was PCR amplified using Pea cDNA library as template. Amplified product was cloned into primary vector pGEM-T easy and confirmed by colony PCR and restriction digestion (Additional file 1A). To check the authenticity of insert, PschSOD clones were sequenced and matched for any mutation. Later, for heterologous protein expression, PschSOD gene was cloned into pET-28a vector (Additional file 1A and B). After confirming the right clone, PschSOD -pET-28a was transformed into protein expression strain i.e. BL21 (DE3). Transformed colonies obtained were checked for protein induction by IPTG (1 mM).

### Growth culture condition optimization for overexpression and purification of protein

The production of properly folded native protein in *Escherichia coli (E. coli)* is often challenging due to aggregation of the overexpressed protein into inclusion bodies. Nevertheless, formation of recombinant proteins in soluble fraction (periplasmic and cytoplasmic) is favored at lower temperature [23]. In this case, the formation of recombinant protein was relatively higher at 37°C (induction period-5 h) as compared to 18°C (induction period-20 h), but a large amount of protein goes to inclusion body at 37°C (Additional file 2A-C, Table 1A). However, at 18°C most fraction of the protein remains in soluble fraction. When induced at 37°C, the amount of native (cytoplasmic or soluble) PschSOD protein, obtained with the purification procedure was ~ 2.8 mg L^−1^ of culture volume with bacterial wet biomass was about ~ 5 g L^−1^, while induction at 18°C resulted in ~ 4 mg L^−1^ of culture volume with bacterial wet biomass was about ~ 4.5 g L^−1^ (Table 1A). Therefore, 18°C was selected for further expression study and overexpression of the protein.

**Table 1:** **Expression and activity of PschSOD in recombinant BL21 cells with variable parameters**

**A: Temperature optimization for IPTG induced bacterial expression of PschSOD**					
While monitoring effect of temperature, Cu or Zn was not added in the growth media during induction period					
Induction temperature (°C)	Induction period (Hour)	Final cell density (g L^−1^)	Quantity of protein^#^ (mg L^−1^)	SOD activity mg^−1^ of crude protein	SOD activity mg^−1^ of purified protein^ψ^
37	5	5	2.8	110	5,000-8,000
37	20	8	3.0	109	5,000-8,000
18	5	3	1.4	117	5,000-8,000
18	20	4.5	3.2	118	5,000-8,000
**B: Effect of Cu (CuSO**_**4**_ **) and Zn (ZnSO**_**4**_ **) supplementation on expression and activity of PschSOD at 18°C**					
Final concentration of CuSO_4_ (μM)	Final concentration of ZnSO_4_ (μM)	Final cell density (g L^−1^)	Quantity of protein^#^ (mg L^−1^)	SOD activity mg^−1^ of crude protein	SOD activity mg^−1^ of purified protein^ψ^
0	0	4.5-5.0	3.2	118	5,000-8,000
0	250	4.5-5.0	3.6	120	5,000-8,000
250	0	4.5-5.1	3.5	790	46,000-56,000
250	250	4.5-5.1	4.0	820	49,000-58,000

Effect of temperature and Cu/Zn supplementation during induction period is summarized. In all the cases, 1 mM of IPTG was used for induction. The protein was purified only from the soluble fraction (cytoplasmic) while inclusion body fraction was not considered. The results presented here represent average of 3 independent protein preparations.

^#^The purified PschSOD protein obtained with a stepwise combination of Ni-NTA affinity chromatography, desalting and buffer exchange.

^ψ^SOD activity of purified PschSOD as measured by pyrogallol assay.

The prosthetic copper and zinc are essential component of CuZn-SODs. Therefore, the effect of copper (Cu) and zinc (Zn) on the expression of PschSOD was investigated by exogenous supplementation of Cu (CuSO_4_) and Zn (ZnSO_4_) in the culture medium following induction with IPTG. The added concentration (250 μM) of each of these compounds does not interfere with the viability of bacterial cells as reflected in OD600 and CFU counting (Additional file 3: Figure S3A and B). Although, supplementation of copper has no effect on the overexpression of PschSOD in soluble form but zinc results in the increase in protein expression (Additional file 4: Figure S4A and B, Table 1B). However, addition of copper causes a nearly 10-fold increase of SOD activity, as observed in both crude extract and Ni-NTA purified protein samples (Additional file 4: Figure S4C and D, Table 1B). Thus, the concentration of both copper and zinc used here is not toxic to the recombinant BL21 cells and can be suitably used to over express PschSOD. It has been previously suggested that, copper and zinc supplementation in growth media can cause transcriptional and translational activation of CuZn-SOD, when overexpressed in bacteria, yeast and plants [16,17,24].

The purified recombinant protein was obtained with a stepwise combination of Ni-NTA affinity chromatography, desalting and buffer exchange with Zeba desalting columns [25]. The homogeneity of the purified proteins was examined on 15% SDS-PAGE. Copper and zinc quantity as determined from ICP-MS, Zincon and Bathocuproine were found to be 1.84 ± 0.15 and 1.6 ± 0.12 respectively per dimer of the recombinant PschSOD as compared to 1.92 ± 0.10 and 1.9 ± 0.09 for bovine CuZn-SOD. The results of the analysis of metal content by ICP-MS, Zincon and Bathocuproine protocol has been shown in Table 2. There is no significant Fe/Mn/ Ni present in the recombinant PschSOD. ICP-MS analysis shows that copper and zinc content was found to be 1.84 ± 0.15 and 1.6 ± 0.12 respectively per dimer of the recombinant PschSOD as compared to 1.92 ± 0.10 and 1.9 ± 0.09 for the commercially available bovine CuZn-SOD. Copper content of PschSOD determined by Zincon and Bathocuproeine was found to be 1.82 ± 0.01 and 1.80 ± 0.08 respectively as compared to 1.92 ± 0.05 and 1.92 ± 0.06 for bovine SOD.

**Table 2:** **Measurement of Copper (Cu) and Zinc (Zn) content of PschSOD by ICP-MS, Zincon and Bathocuproeine protocol**

**Proteins**	**ICP-MS**		**Zincon**		**Bathocuproeine**	
	**Cu**	**Zn**	**Cu**	**Zn**	**Cu**	**Zn**
Bovine CuZn-SOD	1.9 ± 0.046	1.89 ± 0.05	1.92 ± 0.05	----	1.92 ± 0.06	----
PschSOD	1.84 ± 0.15	1.6 ± 0.12	1.82 ± 0.01	----	1.80 ± 0.08	----

The values have been depicted as the quotient of concentration of metal ions to protein (dimer). The data presented here represents metal content from 3 independent protein preparations. There is no significant amount of Fe/Mn/ Ni or any other metal was found in the analysis.

### Determination of native and subunit molecular weight

**Figure 1:**
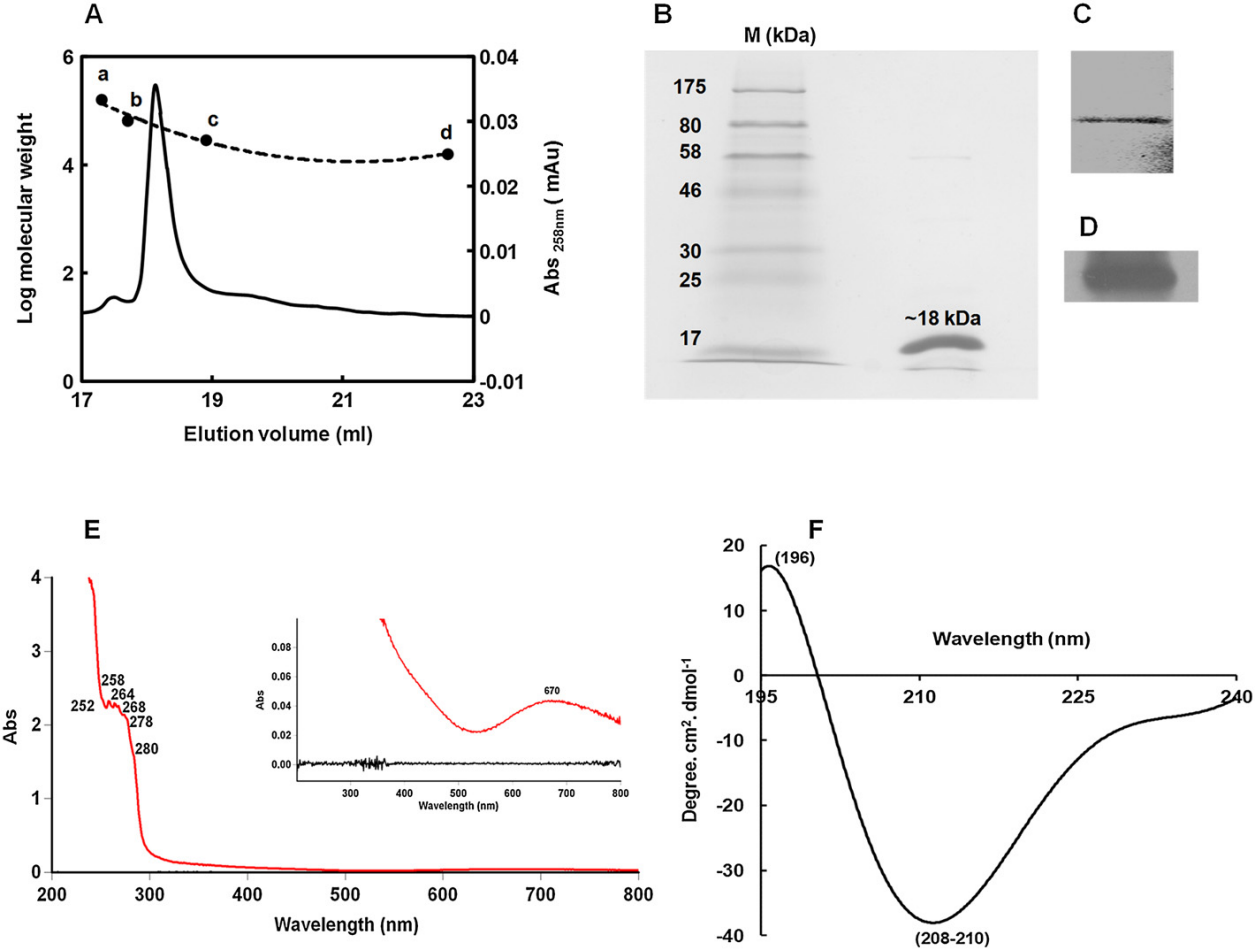
**Molecular mass of purified PschSOD and spectral analysis. (A) **Native molecular mass of recombinant PschSOD was measured with respect to standard protein marker as resolved in Biosil-250 analytical gel filtration column. Standard proteins used were: (a) 160 kDa-Glucose oxidase (b) 66.5 kDa-BSA fraction V (c) 29 kDa-Carbonic anhydrase and (d) 16 kDa-Bovine CuZn SOD (monomer) respectively **(B)**. The 15% SDS–PAGE showing PschSOD subunit. Subunit molecular mass as calculated using the molecular standard markers (M)suggest that the subunit mass of the protein is ~ 18 kDa.Western blot taking1μg of the protein and probed with poly-his antibody has been shown in **(C)** while probed using CuZn-SOD specific antibody has been depicted in **(D)**. **(E)** Room temperature (25°C) UV-Visible electronic transition spectra of 2 mg ml^−1^ PschSOD. The inset depicts the spectral resolution in an extended scale showing prominent broad peak at 670 nm, characteristics of d-d transition for copper in CuZn-SODs. CD spectral analysis of the protein at 25°C is represented in **(F)**. Negative band at 208 nm region and positive band at 196 nm is shown which suggest abundance of *β*-sheet in the protein.

Gel filtration chromatography of PschSOD shows a distinct single retention profile, corresponding to 36–38 kDa molecular mass (Figure 1A). However, the 15% SDS-PAGE analysis suggest that the sub-unit size PschSOD is ~18 kDa (Figure 1B). Western blot analysis by using both monoclonal anti-His antibody and polyclonal anti-CuZn-SOD, confirms the presence of Histidine tag in recombinant protein corresponding to the ~18 kDa molecular marker (Figure 1C and D respectively). These results suggest that the recombinant his-tagged PschSOD maintains its dimeric form in native condition during its recombinant synthesis.

### Spectroscopic characterization

The recombinant PschSOD, display multiple electronic transition maxima referring to phenylalanine transitions at 252, 258, 264 and 268 nm and a shoulder at 278–280 nm (Figure 1E). These characteristics absorptions may be indicative of reduced quantity of tyrosine and tryptophan residues, as reported for other plant CuZn-SODs [6,7,26]. Deduced amino acid sequence from the cDNA, also corroborate this argument, where a single tyrosine residue is present at 98th position.

The spectrum in the visible region showed an electronic transition at around 670 nm (Figure 1E inset). Although, the maxima of d-d transition for Cu^2+^ co-ordination of chloroplastic CuZn-SOD is generally peaks at 680 nm [6], the 10 nm blue shift of this maxima in recombinant PschSOD suggest that the ligand environment of the prosthetic copper ion is different, resembling the cytosolic type of CuZn-SOD in its ligand to protein interaction.

Circular Dichroism (CD) spectrum of PschSOD (Figure 1F) was analyzed in 190–240 nm regions. A prominent negative band at 208–210 nm regions was discernible indicating the presence of small amount of α- helix but with high percentage of *β*-sheet content similar to human and other eukaryotic SODs [6,27]. CD spectra of chloroplast and cytosol CuZn-SODs from spinach also show similar spectral resolution [28]. Thus, it appears that the PschSOD conserves the *β* -barrel structure of the CuZn-SODs in its recombinant form.

### Measurement of PschSOD superoxide dismutase and peroxidase activity

**Figure 2:**
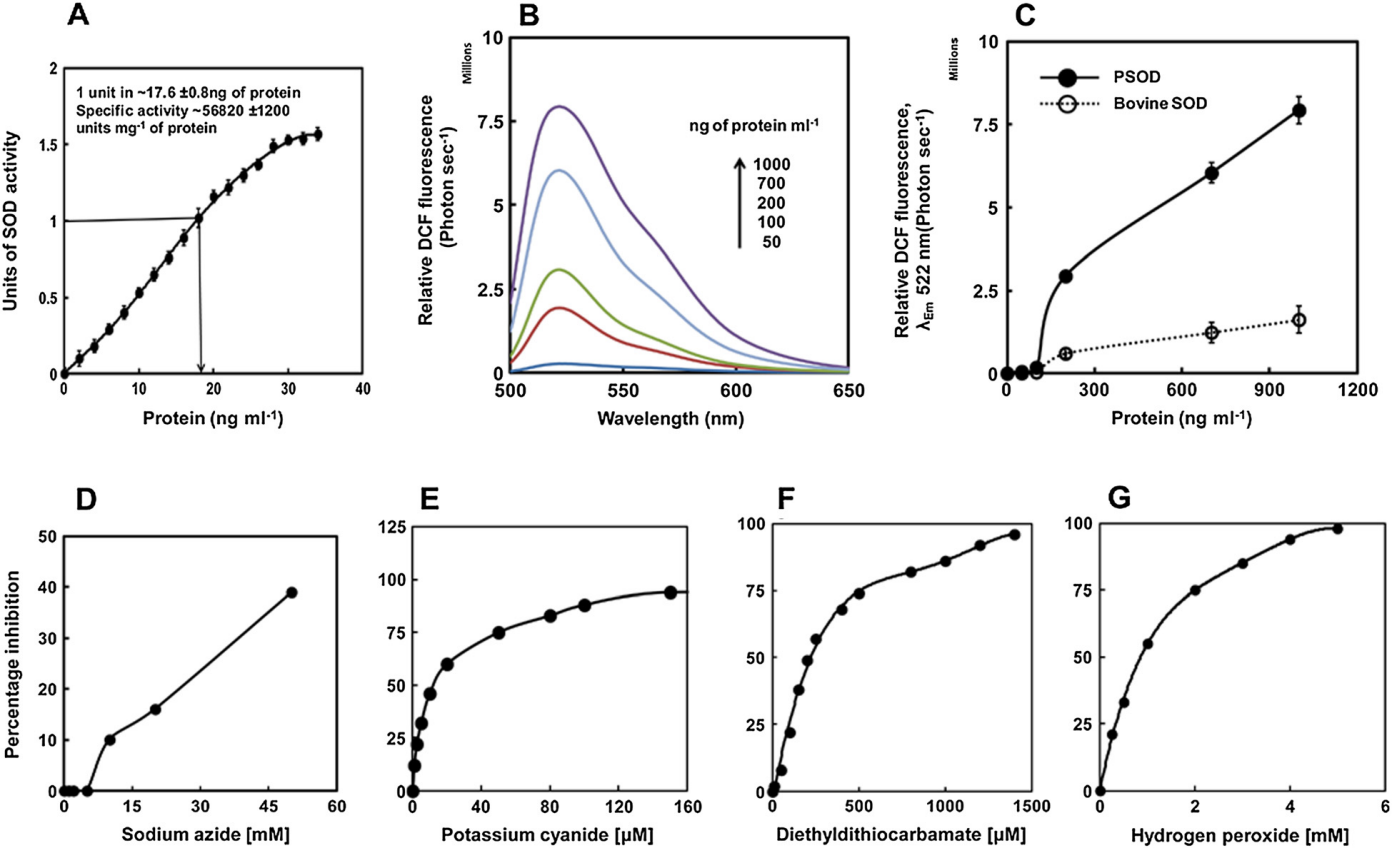
**Measurement of superoxide dismutase (SOD) and peroxidase activity of PschSOD and effect of inhibitors on SOD activity. (A)** Evaluation of SOD activity by pyrogallol assay with increasing protein concentrations of PschSOD. Specific activity (units/mg) of the protein was calculated in the linear range of superoxide scavenging activity and displayed in the figure inset. **(B)** Peroxidase activity of PschSOD measured as increase in relative 2′, 7′-dichlorfluorescein (DCF) fluorescence with increasing protein concentration (as mentioned in the figure). Comparative assessment of peroxidase activity with bovine CuZn-SOD is represented as the relative DCF fluorescence at emission maxima (λ_max_-522 nm) under 480 nm excitation in **(C)**. Closed circle (●) represents the PschSOD while open circle (○) with solid line represents bovine SOD. Inhibitors tested were **(D)** Sodium azide **(E)** Potassium cyanide **(F)** Diethyldithiocarbamate and **(G)** Hydrogen peroxide. The residual SOD activity was determined taking 1 unit of SOD as 100%. Potassium cyanide and diethyldithiocarbamate acts immediately on CuZn-SOD while for sodium azide and hydrogen peroxide, PschSOD was incubated at desired concentration for 20 min at 37^°^C and assayed for residual activity.

Superoxide dismutase specific activity of PschSOD, assayed by pyrogallol method was calculated to be 45,000-56,000 unitsmg^−1^ of protein (Figure 2A); an activity not reported in literature hitherto. This span of variation was noted from four different individual preparation of the recombinant protein (45, 520; 47, 320; 49, 220; 56, 103 units mg^−1^ protein). On comparative basis, the observed activity was recorded to be nearly 6-fold higher than the activity reported for commercial protein (Sigma-S8160). In addition to the usual superoxide dismutation activity, CuZn-SOD has also a peroxidative function, where the enzyme utilizes its own dismutation product, the H_2_O_2_ in presence of bicarbonate [25]. Bicarbonate mediated peroxidase activity was measured as increase in 2′, 7′ dichlorfluorescein (DCF) fluorescence intensity with increasing concentration of protein and compared with the activity obtained using commercial CuZn-SOD (Figure 2B and C). The peroxidase activity of the enzyme was also found to be nearly 5-fold higher than the commercial bovine CuZn-SOD (Sigma-S8160).

### Inhibitor studies on PschSOD activity

Results on the effect of various inhibitors on the PschSOD activity have been shown in Figure 2D-G. The recombinant enzyme was least affected by Sodium azide (NaN_3_), as only 40% of native activity was inactivated by 50 mM Potassium cyanide (KCN). Addition of 5 mM of Hydrogen peroxide (H_2_O_2_) completely inhibited of the enzyme activity. KCN was also able to inhibit 100% of the activity at very low concentration (160 μM), while Diethyldithiocarbamate (DDC) could completely abolish the enzyme activity at 1400 μM.

In general, the CuZn SODs purified from various sources exhibit a wide range of susceptibility to inhibitors like NaN_3_, KCN and H_2_O_2_ [29]. DDC and KCN are specific inhibitors for CuZn-SODs and required relatively lesser concentrations to cease enzyme activity and the inhibition is also instant. However, NaN_3_ and H_2_O_2_ mediated inhibition is slow and requires higher concentrations for complete inhibition.

### Effect of pH and temperature on activity and stability of PschSOD

**Figure 3:**
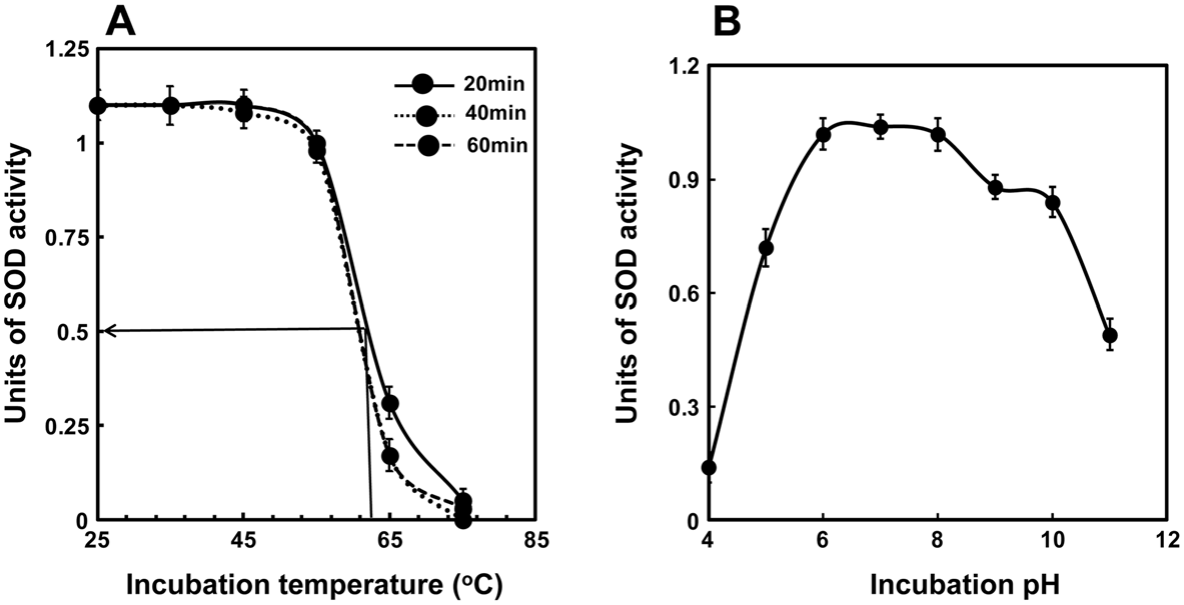
**Effect of incubation temperatures and pHs on the PschSOD activity.** Twenty units equivalent of protein was incubated at different temperatures (25°C-75°C) for different time interval (20, 40 and 60 min) as shown in **(A)** and the remaining activity was assayed. The T_1/2_ of the activity for 60 min has been marked with the solid line. Effect of different pH on the activity of PschSOD superoxide dismutase activity has been shown in **(B)**. 20 units equivalent of protein were incubated in 50 mM of different buffer with respective pH at 37°C and the residual activity was assayed.

The effect of incubation temperature on PschSOD activity was studied and the results are shown in Figure 3A. The enzyme activity was found to be temperature resistant till 55°C, beyond which the activity declined sharply. The time kinetics of alternation in activity profile was also monitored at selected temperatures. The t_1/2_ (reduction in activity to 50%) was calculated to be 62°C, which remains nearly comparable for 20, 40 and 60 min incubation. The enzyme activity showed a broad range of pH sustainability (Figure 3B). The optimal activity of the enzyme was found to be in the pH range of 6–8.0. About 70% of its optimal activity was retained in the acidic pH of 5.0 and in the alkaline pH of 10.0. While 90% of the maximal activity was lost at lower pH (pH-4.0), the remaining activity at pH 11.0, as compared to maximal was nearly 40%. Although, it is suggested that the CuZn-SOD is not pH dependent [30], but recent results from recombinant proteins came with contradicting result [31-33]. The decrease of the enzyme activity at acidic pH might be due to the dissociation of dimer into monomer, as the acidic pH favors the monomer formation, whereas the alkaline pH favors the dimer formation [34]. Charge interaction between the subunits has been also postulated to be a major determinant of CuZn-SOD activity at varying pHs [34].The lowering of activity at acidic pH in this group of SODs is also attributed to the release of Cu ion from the active site [25].

### Effect of denaturating and proteolytic agents on PschSOD

**Figure 4:**
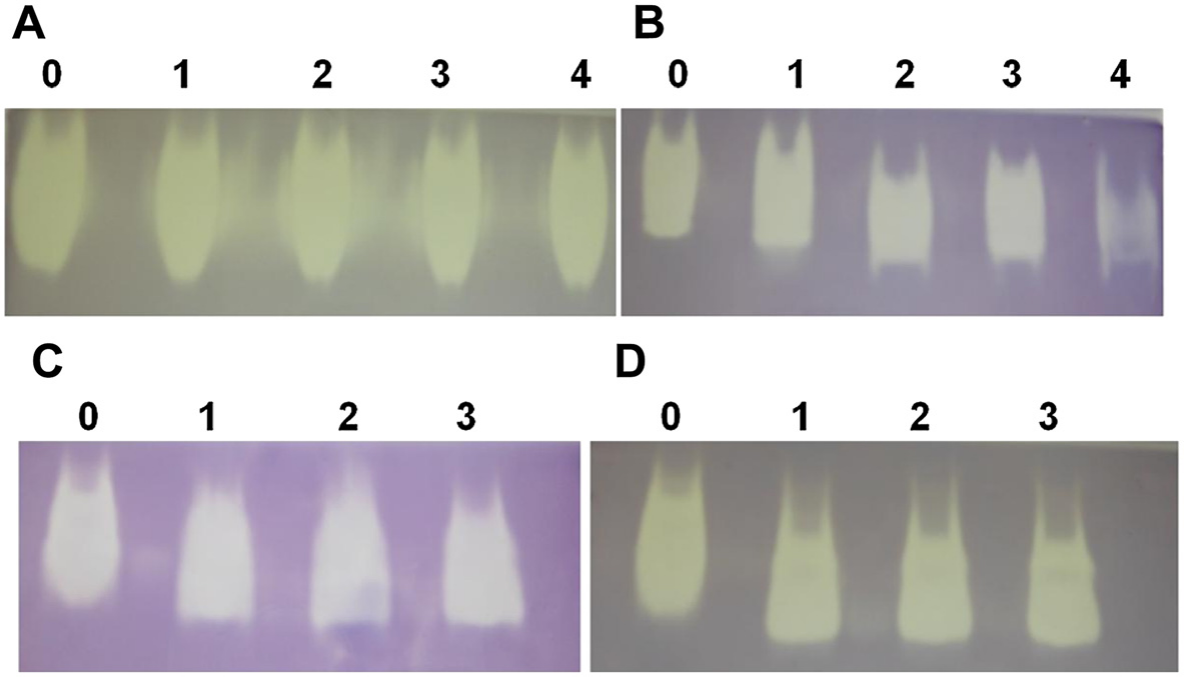
**Effect of incubation of PschSOD with denaturating and proteolytic agents.** PschSOD was incubated in the presence of **(A)** Urea **(B)** Imidazole at concentration 1–4 M as mentioned in the figure and with proteolytic enzyme **(C)** Trypsin (1/20 w/w) and **(D)** Chymotrypsin in 50 mM potassium phosphate buffer, pH 7.8, at 37^°^C with varying time intervals of 0–3 h. Residual activity was analyzed on 12% native-PAGE with NBT staining as described in method section. The densitometric analysis of the *in gel* SOD bands has been represented as Additional file 3.

PschSOD exhibited very high tolerance to denaturating and proteolytic agents. The activity was not significantly affected by 4 M of urea and imidazole (Figure 4A-B, Additional file 3A). The catalytic function of the enzyme remains unaltered by incubating the protein with trypsin and chymotrypsin for 3 hour (Figure 4C-D, Additional file 3B).

Proteolysis of proteins is often used as a selection tool to assess protein stability and to probe its energy states [35]. Extensive characterization of different prokaryotic and eukaryotic CuZn-SODs has established that monomeric and dimeric CuZn-SODs have very different activity, thermal stability, metal affinity, and resistance to proteases [36]. Various factors responsible for imparting high stability are relatively small solvent-exposed surface area, *β*-barrel fold, tight hydrophobic dimer interface and extensive ion-pair networks and better salt bridge formation [13]. Results of proteolytic susceptibility of PschSOD further suggested that the protein has limited access to partially and globally unfolded conformations under native conditions and the resistance might have been due to minimized occurrence of accessible conformations susceptible to proteolytic attack [37].

### Longevity of PschSOD stability and activity

**Figure 5:**
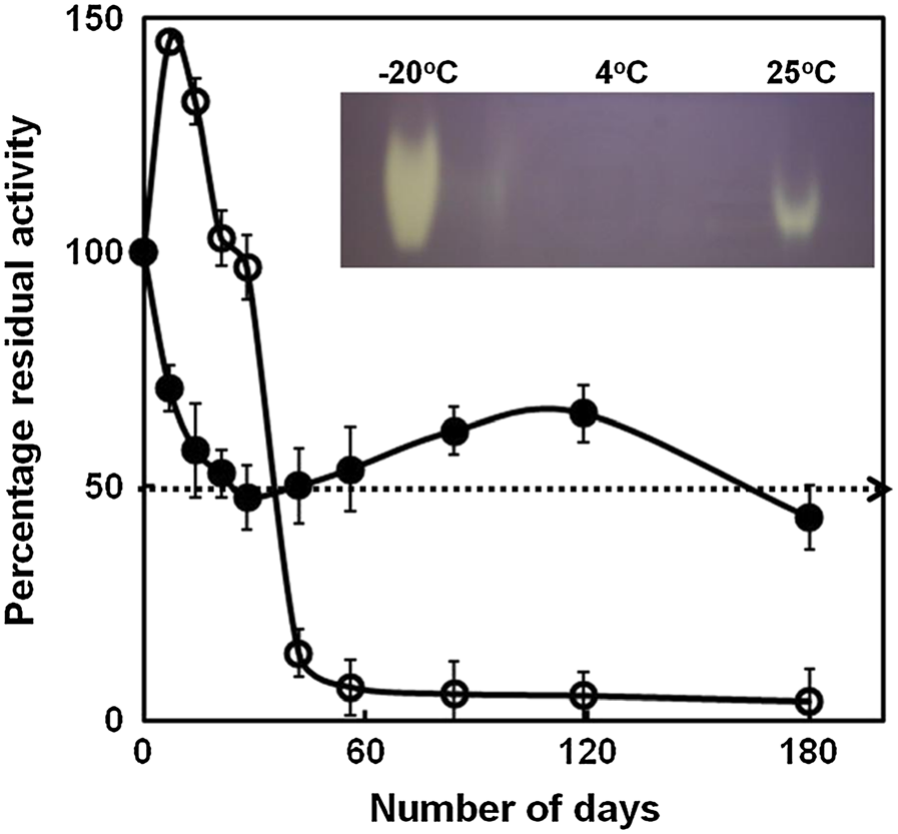
**Longevity of PschSOD activity.** Purified PschSOD was tested for its longevity both at room temperature and 4°C. Percentage residual activity was judged periodically with pyrogallol assay and further analyzed with native-PAGE and SOD specific staining after 180 days. The protein sample kept at −20°C was also judge for native activity and taken as control because its remains unaltered even after 180 days. Closed circle (●) represents the sample kept at room temperature while open circle (○) with solid line represents enzyme sample kept at 4°C.

Purified PschSOD was tested for its longevity through storage at room temperature (25°C) and 4°C and the protein samples kept at −20°C were considered as control (Figure 5). The enzyme did exhibit only 50% loss in activity even after 180 days of storage at room temperature. However, at 4°C the protein showed a transient elevation in activity, but undergoes a rapid inactivation in subsequent days. The final loss in activity is accounted for about 90% of activity as compared to activity of protein stored at −20°C. Higher reduction of PschSOD activity at 4°C suggests that the protein is not stable at this temperature and use of some cryo-protectants and/or suitable protease inhibitor may be able to maintain its original activity. The activity of the protein samples following 180 days of storage at different temperatures were also evaluated *in-gel* activity assay and the results have been shown as inset to Figure 5 inset.

### PschSOD sequence similarity and divergence

**Figure 6:**
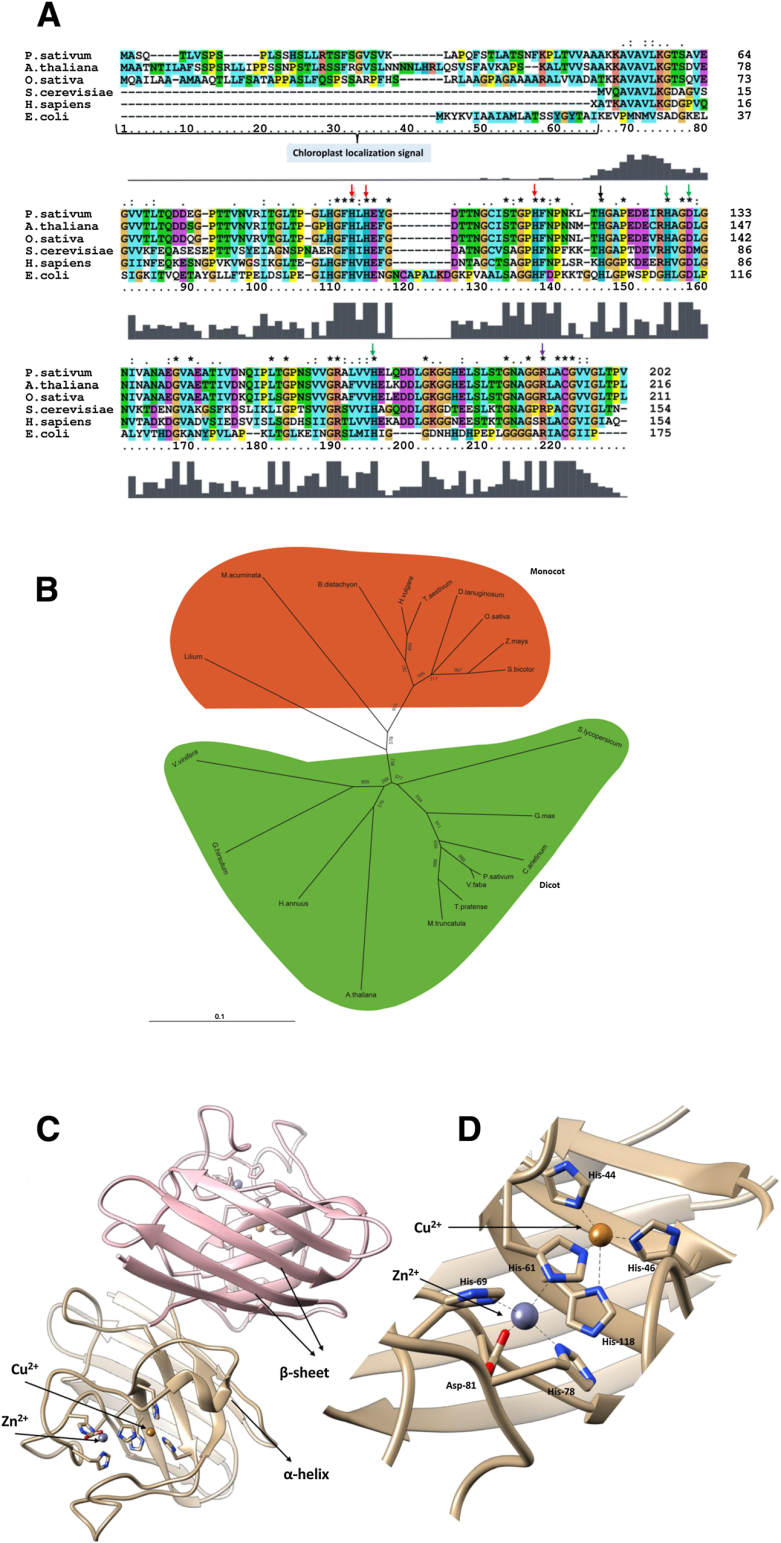
**Sequence analysis and Homology modelling of PschSOD (A)** Multiple sequence alignment was performed using ClustalW software (http://www.ebi.ac.uk/Tools/msa/clustalw2/). Accession no. of corresponding gene sequence are: *Pisum sativum*: emb|CAA39819.1|, *Arabidopsis thaliana*: gb|AAD10208.1|, *Saccharomyces cerevisiae*: ref|NP_012638.1|, *Homo sapiens*: pdb|1L3N|A and *Escherichia coli*: ref|WP_000875886.1|). Various conserved residue (His-44, His-46, His-61, and His-118) for Cu^2+^ and (His-61, His-69, His-78, and Asp-81) for Zn^2+^ has been marked with red and green arrow. Common Histidine (His-61) is also marked by black arrow while positively charged Arg-141 is marked by purple arrow. Chloroplast localization signal is present in SOD from plants only. **(B)** Tree was constructed using neighbor-joining method with 1000 bootstrap replicates. Monocot and dicot families are separated in two different clades. **(C)** Homology modelling of PschSOD was prepared by using default parameter at SWISS model workspace (http://swissmodel.expasy.org). The figure shows the dimer of modelled PschSOD with both Cu^2+^ and Zn^2+^ in active site. Active site is exposed outside in both monomer. A helix and beta sheets are also labelled. **(D)** All the co-ordinates of Cu^2+^ (Brown) and Zn^2+^ (Black) with His-61 being common in both cofactors show.

The percentage of identity and divergence in the amino acid sequence of selected SOD was identified using Clustal W program (Figure 6A). The results of this analysis showed that PschSOD was closely related to *Arabidopsis thaliana*-SOD sharing 77.23% similarity, while 71.29% similarity was evident for Rice-SOD, respectively. PschSOD also shared about 58% similarity with *S. cerevisiae* and *H. sapiens.* The Cu^2+^ site responsible for catalysis is also conserved as it is coordinated by four His residues (His-44, His-46, His-61, and His-118) and the Zn^2+^ site is bound to the protein through His-61, His-69, His-78, and Asp-81 (Figure 6A) which is also conserved. To understand the evolutionary relationship between SOD proteins from various phyla, an unrooted phylogram was constructed using Clustal X with 1000 bootstrap replicates. From the phylogenetic analysis, we found that plant SOD from various family made clades according to their family. Monocot and dicot fall in two different clades (Figure 6B). Bootstrap values of the nodes were found to be high in various families. A strong bootstrap value support for individual clades added confidence to the cladogram construction.

### PschSOD: motif to structural analysis

In order to detect the conserved domains and motifs among various SODs, multiple sequence alignments were carried out. The N-terminal chloroplast localization signal was observed in all plant SOD, whereas none of the yeast, human and *E. coli* proteins possessed this signal (Figure 6A). Plant SODs were more similar to each other than to human and *E. coli.* The Histidine residue in alignment is conserved in all SODs across the phyla. On C-terminus, positively charged Arg-141 located in the active site channel was also conserved without any exception among all the members included in the presented figure (Figure 6A).

The search for structural homolog was carried out using PschSOD as candidate. PschSOD is a CuZn-SOD; it was modelled against four different CuZn-SODs which showed more than 90% sequence similarity (Expasy SWISS-Homology Modelling). Out of these models, two (3 km2 and 3s0p) were selected on the basis of presence of copper and zinc cofactor. In 3 km2 model, only zinc ions were present, so we also considered 3s0p model as it have copper ion in it. Both the models were superimposed to get both copper and zinc incorporated. Structure of PschSOD revealed 8 β sheets and 2 α helices mostly exposed. All the co-ordinate for Cu^2+^ and Zn^2+^ were found to be conserved. Histidine bound to copper and zinc are highlighted on multiple sequence alignment (Figure 6A). The authenticity of the model was confirmed from various scores like E value (7.6e-68). The color represented Estimated per-residue inaccuracy visualized using a color gradient from blue (more reliable regions) to red (potentially unreliable regions). Overall, the sequence identity is more than 90% and the entire characteristic residue forming the active site is quite conserved throughout the species. However, in the absence of molecular structure, it is hard to explain the structural basis for the enhanced kinetic behavior of PschSOD.

## Conclusions

SODs are critical enzymes that manage oxidative stress in almost all organisms dealing with the inevitable metabolic product of aerobic respiration i.e.O_2_^∙−^ [8]. Plants contain multiple SOD isoenzymes, transcribe through distinct gene families. In plants, three forms of the enzyme exist, as classified by their active site metal ion: copper-zinc, manganese, and iron forms. The distribution of these enzymes has been studied both at the sub-cellular level and at the phylogenic level. It is only in plants that all three different types of SOD are known to coexist [3]. Their occurrence in the different sub-cellular compartments of plant cells allows us to study their molecular evolution and the possibility of understanding why three functionally equivalent but structurally different types of SOD have been maintained? Among all the isoforms of the enzyme, CuZn-SOD is the most abundant in plants and thoroughly dispersed throughout evolution. Phylogenetic analysis of plant sequences show that all chloroplast CuZn-SODs are more related to each other than to cytosolic CuZn-SODs, which also form their separate sub-group [38]. Chloroplast contains both Fe and CuZn-SOD and, distribution of these isoforms vary among different species [39]. Several functions have been postulated for SODs in chloroplasts like protection to oxidative stress (by controlling superoxide and hydrogen peroxide in the water–water cycle and control of free redox-active metals), photoprotection, regulation of electron transport and signaling. It is also remarkable to note that expression of Fe-SOD and CuZn-SOD is found to be reciprocal in response to Cu treatment, different stress treatments and development stages [8,39]. Thus, chloroplastic isoform of the enzyme remains an excellent choice to study kinetic behavior of this class of enzyme to gain deeper insight into its structural feature, which can help the enzyme to modulate its activity to perform these diverse functions. The rationale of the present study is to describe an efficient method for the recombinant expression of *Pisum sativum* chloroplastic CuZn-SOD (PschSOD) in the enzymatically active (soluble) form. The protein was evaluated for its biochemical characteristics and found to possess the highest activity, reported so far. Structural and signature motifs analysis through homology modeling further confirm that PschSOD retains the conserved architecture of this class of protein. But it is the non-conserved sequence compared to other which could be responsible for this high activity of this protein. Merely on the basis of homology modelling and multiple sequence alignment it is hard to elucidate the main character for the high activity of the protein. However, the higher kinetic properties in terms of its catalytic activity as observed in this study needs further understanding on the molecular aspects of substrata accessibly to the active site and its interaction kinetics. Thus, the purified PschSOD can be utilized for its structural study, has application in pharmaceutical industries and highly recommended for producing transgenic plants that need additional tolerance to oxidative stresses.

## Methods

### Plasmids, bacterial strain and reagents

Fresh seed of Pea (*Pisum sativum*) were sown on a 2:1:1 mixture of vermiculite, peat moss and perlite, and were maintained for 3 days in darkness before being transferred to normal growth conditions. Later the plants were grown at 26 ± 2°C temperature under 16 h light and 8 h darkness period. Leaves of 7-day old pea seedling were used to isolate RNA. *E. coli* DH5α was the host strain for plasmid construction and cloning. Plasmid vectors used for cloning were pGEM-T easy (Promega) and pET-28a (Novagen). Unless indicated, all analytical grade reagents were procured from Sigma. The expression vector pET-28a, *E. coli* BL21 (DE3) cells were obtained from Novagen. Ni-NTA columns and matrix were procured from Qiagen. Chromatography columns were procured either from Amersham Pharmacia or Pierce.

### Construction of pea cDNA library and Cloning of *SOD* gene from pea

Pea cDNA library was constructed as described earlier [40]. In our recent study, PschSOD was found to be an interacting partner of PDH45 [41]. Complete sequence of this PschSOD matched with earlier reported SOD from pea (NCBI Accession no. CAA39819). Gene specific primers were designed using Primer 3 software. *NdeI* restriction site was inserted prior to PschSOD F (5′CATATGGCTGCCAAGAAAGCCGTCGCTG3′) and *BamHI* restriction site was inserted after PschSOD R (5′GGATCCTTATACTGGAGTCAAGCCAACC3′). Using pea cDNA library as template, PCR reactions were carried out in a final volume of 50 μl containing 5 μl 10X taq buffer (Agilent Technologies); 200 μM of dNTPs; 0.2 ng of each primer; 5 units of Taq DNA polymerase (Agilent Technologies). The PCR program used was as follows: 95°C for 5 min (1 cycle), 94°C for 30 s, 58°C for 30 s, 72°C for 1 min (32 cycles), and 72°C for 10 min (1 cycle). PCR product was separated on agarose gel. The expected band was excised from gel and purified using Midi gel extraction column (Advanced Microdevices Pvt. Ltd.). Using TA cloning vector, insert was cloned in pGEM-T easy vector. Confirmation of primary cloning of PschSOD -pGEM®-T by colony PCR and restriction digestion is shown in Additional file 1: Figure S1A.

### Construction and transformation of the recombinant plasmid (pET-28a-PschSOD) for heterologous expression in *E. coli* system

PschSOD-pGEM-T clone and pET-28a vector were digested with both *NdeI* and *BamHI* restriction enzyme. Later, PschSOD -insert and pET-28a backbone were ligated and transformed in *E.coli* DH5α. Colony PCR and restriction digestion confirmed the final cloning of PschSOD in pET-28a expression vector. For heterologous protein expression, PschSOD -pET-28a clone was transformed in BL21 (DE3) cells. Transformed colonies obtained were checked for protein expression with the help of IPTG (1 mM).

### Overexpression of PschSOD in BL21 bacterial cells

For overexpression of PschSOD, the overnight culture of recombinant BL21 (DE3) cells harboring pET-28a- PschSOD plasmid was diluted to 100 times in LB media, supplemented with 50 μg/ml kanamycin on an orbital shaker (240 rpm) at 37°C. When the OD_600_ of the culture reached a value of 0.4-0.6, IPTG was added at a final concentration of 1 mM and culture was grown for another 20-h at 18°C. Total bacterial proteins were fractionated into periplasmic, cytoplasmic and inclusion body fractions, following published protocol [42] and the presence of recombinant protein in soluble and/or insoluble fraction was checked on 15% SDS-PAGE taking 20 μg of protein and bands were visualized by staining with PhastGel Blue R(Amersham Biosciences, Sweden) and western blotting was done using Penta-His (Qiagen) and CuZn-SOD (Agrisera) antibody, by the process described earlier [43].

### Optimization of the culture condition for PschSOD solubility, production and activity

The effect of temperature and copper-zinc supplementation on recombinant PschSOD overexpression and activity was examined. To check the solubility of PschSOD, growth temperature during induction period was maintained at 37°C and 18°C. Twenty hour post induction, cells were harvested by centrifugation (8000 × g for 20 min at 4°C) and fractionated as described earlier [42]. Both copper (CuSO_4_) and zinc (ZnSO_4_) were supplemented at final concentration of 250 μM in growth medium during induction period at 18°C. SDS-PAGE, western blot and activity measurement of the crude extract and purified protein (methods discussed in later sections) were performed. Other details are provided in the figure legends. Effect of copper-zinc supplementation on bacterial viability was also checked during induction period by taking OD at 600 and counting the colony forming units (CFU) [44]. Cultures with identical treatment, but without IPTG induction served as negative controls (non-induced).

### Purification of the recombinant PschSOD

To purify the proteins in native confirmation, induced culture (supplemented with 250 μM of both CuSO_4_ and ZnSO_4_) was harvested by low speed centrifugation (8000 X g, 20 min), and the protein was affinity purified using Ni-NTA Fast Start columns (Qiagen) with slight modification from manufacturer’s instructions. Briefly, the pellet was re-suspended in lysis buffer containing 50 mM NaH_2_PO_4_, 300 mM NaCl, 10 mM imidazole, 1 mg/ml lysozyme, 25 unit/ml of Benzonase Nuclease and protease inhibitor cocktail for histidine tagged proteins (Sigma).The suspension was further sonicated on ice to lyse the cells (six times for 10s each time with 5 s pause between). The clear lysate was loaded into the Ni-NTA column, and washed with optimized volume of wash buffer containing 50 mM NaH_2_PO_4_, 300 mM NaCl, 20 mM imidazole. The enzyme was eluted with elution buffer with 250 mM imidazole. The enzyme sample was desalted and buffer exchanged with low ionic strength phosphate buffer (5 mM K-PO_4,_ pH-7.2) using Zeba Desalt Spin Columns (Pierce) and further dialyzed for 48-h (12 hour periodic buffer change) with the same buffer at 4°C and concentrated using Amicon centriprep YM-10 centrifugal filter devices (Millipore).

Purity of the protein sample was analyzed on 15% SDS-PAGE taking 1 μg of protein and proteins bands were visualized by staining with PhastGel Blue R (Amersham Biosciences, Sweden) and polyhistidine tag was confirmed with western blotting using Penta-His antibody (Qiagen).

Protein concentration was determined according to Bradford method, by using bovine serum albumin (BSA) as the standard [45]. Metal content of the protein was measured by ICP-MS at SGS India Pvt. Ltd. Gurgaon, India and further analyzed following Zincon and Bathocuproine protocols [46-48]. In all the cases, metal content were analyzed from proteins that were desalted and buffer exchanged with 5 mM K-PO_4_ buffer (pH-7.2). The evaluation was made using Cu and Zn content of bovine CuZn-SOD (Sigma-S8160) as reference standard and represented as the molar quotient of the metal ions and protein.

### Determination of molecular weight (M_r_ and subunit size)

The native molecular weight of the purified PschSOD was determined by analytical gel filtration chromatography on a Bio-Sil 250 column (Bio-Rad). 5 mM K-PO_4_ (pH-7.2) buffers containing 150 mM NaCl was used. The column was calibrated with the standard proteins: Glucose oxidase, BSA fraction V, Carbonic anhydrase and Bovine CuZn SOD (monomer). Subunit size was determined by 15% SDS-PAGE, using NEB’s pre-stained protein ladder.

### Spectroscopic measurement

UV–vis absorbance spectra were recorded from 800 to 200 nm with Varian Cary 300 Bio double - beam spectrophotometer using a cell with a 10 mm path length, at 25°C. The concentrations of SOD samples were 1 mg ml^−1^ in 5 mM K-PO_4,_ pH-7.2.

### Spectropolarimetry

CD experiments were conducted in a J-815 Circular Dichroism Spectropolarimeter (JASCO International Co., Ltd. JAPAN) at 25°C using a quartz cuvette with a 0.1-cm path length. Spectra from three scans from 190 to 260 nm at 30 nm/min were averaged, and the buffer baselines were subtracted from their respective sample spectra. Measurements of the molar ellipticity were calculated following equation: [θ] _MRW_ =100⋅θ⋅10^−3^ /C_mr_⋅0.1. Where [θ] _MRW_ is the mean residue weight in degrees, C_mr_ represents the molar concentration multiplied by the number of amino acids, and 0.1 is the path length in cm. The final protein concentration of each sample used in the measurements was quantitated using a Bradford Assay kit and shown to be 0.2 mM.

### SOD activity staining and assay

Native PAGE and specific staining for SOD were performed as described previously [49]. Briefly, each sample was separated by electrophoresis on 12% polyacrylamide gel and then the gel was first soaked in 1.23 mM NBT for 20 min, briefly washed, then soaked in the dark in 100 mM potassium phosphate buffer (pH 7.0) containing 28 mM N, N, N′, N′-tertamethylethylenediamine (TEMED) and 2.8 × 10^−2^ mM riboflavin for additional 20 min. The gel was briefly washed and illuminated on a light box for 15 min to initiate the photochemical reaction. All the procedures were carried out at room temperature and the SOD bands were visualize as clear white region in the dark formazon background.

SOD activity was assayed based on inhibitory effect of SOD on the spontaneous autoxidation of pyrogallol [50]. One unit of SOD was defined as the amount of protein required inhibiting the initial rate of pyrogallol autoxidation by 50% and was expressed as units/mg of protein. The generation of O_2_^∙−^ radical (Δ Absorbance min^−1^- 0.20) was kept constant throughout the experiment. Concentration dependent inhibitory effect of various known inhibitors of CuZn-SODs like NaN_3_, KCN, H_2_O_2_ and DDC were examined. KCN and DDC, the inhibitory effect of NaN_3_ and H_2_O_2_ was observed only when the enzyme samples (equivalent to 5 units) were pre-incubated for 20 min at different concentrations of inhibitors and assayed with 1 unit equivalent of protein.

### Measurement of bicarbonate mediated peroxidase activity

Bicarbonate mediated peroxidase activity of enzymes were measured according to published procedure [51]. Briefly, 2, 7-Dichlorfluorescein-diacetate (DCFH-DA) was first dissolved in methanol by vortexing and then hydrolyzed by 0.01 M NaOH in dark for 30 min at room temperature. Peroxidase activity was measured at increasing protein concentration in 100 mM K-PO_4_ buffer, pH 7.4 containing 100 μM DTPA, 15 μM DCFH and 25 mM NaHCO_3_ at 25°C. The reaction was initiated by adding 300 μM of H_2_O_2._ The reaction cocktail was incubated for 5 minutes, and the net peroxidative activity was measured as increase in 2, 7-Dichlorfluorescein (DCF) fluorescence emission intensity at 522 nm under 480 nm excitation at 25°C (excitation and emission slit width was maintained at 5 nm each).

### Effect of temperature and pH on SOD activity

Thermostability of the protein was studied by incubating the enzyme samples at different temperature (25°C - 95°C) in 5 mM K-PO_4,_ pH-7.2. Aliquots required for the assay were removed at different time intervals and kept immediately on ice, for the determination of residual enzymatic activity.

The effect of pH on the stability and enzymatic activity were examined by incubating protein samples in 50 mM buffers at different pH values (4–12) (pH 4.0-6.0, citric-citrate; pH 7.0-8.0, potassium phosphate; pH 9.0-12.0 glycine-NaOH) for 30 min. The pH of the incubation mixtures was measured after the addition of the enzyme and also following different time intervals. The activity of the samples was assayed as described in earlier section.

### Effect of denaturating and proteolytic agents on PschSOD activity

In order to study the effect of proteolytic enzymes, the enzyme was incubated with 1/20 w/w of trypsin and chymotrypsin in 50 mM K-PO_4_ buffer, pH 7.8, at 37°C for defined time intervals. For chymotrypsin digestion, CaCl_2_ was added to a final concentration of 20 mM. Similarly, defined concentration of denaturating agents; urea and imidazole (1-4 M) was added to the protein sample, incubated in 50 mM K-PO_4_ buffer, pH 7.8, at 37°C for 1 hour and evaluated for its residual activity. Control samples were also incubated under identical conditions without the proteolytic enzyme/denaturating agents. Aliquots were removed at specified time intervals and analyzed for activity on 12% native-PAGE and stained for SOD activity. *In gel* SOD activity bands were also analyzed by quantity one software (Biorad) and represented in graphical format (Additional file 5A and B).

### Effect of long-term storage on PschSOD stability and activity

PschSOD suspended in 5 mM K-PO_4_ buffer, pH 7.2 was evaluated for its stability and activity under long-term storage at 25°C for 180 days. The measurement was made periodically with pyrogallol assay system. Control enzyme sample was incubated under identical conditions at 4°C. After 180 days both the enzyme sample were also evaluated for their *in gel* activity.

### Multiple sequence alignment

Amino acid alignment of 5 CuZn-SODs from various organisms with several Histidine (His) signatures are shown. The conserved SOD motifs are indicated by black box on aligned sequences. The alignment was made using ClustalW software (http://www.ebi.ac.uk/Tools/msa/clustalw2/) [52] on entire protein sequence for each SOD type. CuZn-SODs are identified on left and numbers of amino acid residues are given on right. Asterisks indicate conserved amino acids while dots represent conservative amino acid changes. Gaps in the amino acid sequences are introduced to improve the alignment. An un-rooted NJ tree is made from the whole protein sequences of various plant CuZn-SODs. Tree was made using clustalX1.81 and viewed in Treeview 1.6.6 software. Analysis of the Phylogenetic tree revealed that various CuZn-SOD is divided into different classes i.e. monocot and dicot, each represented by a clade. CuZn-SOD from various genera belonging to same class falling in the same clades based on the bootstrap support value ≥50%. Scale bar represents 0.1 amino acid substitutions per site.

### Protein analysis: Homology modelling of PschSOD

Homology modelling of PschSOD was prepared by using default parameter at SWISS model workspace (http://swissmodel.expasy.org/) [53,54]. We used automated mode for modelling as it selects desired template itself on the basis of highest similarity against the primary amino acid sequence. Templates used for modelling are pdb-id: *3 km2.2, 3hog.1, 3s0p.1, 1srd.1*. Each model generated was checked for various parameters which include Z score, GMQE (global quality estimation score) and QMEAN (model quality estimation) score to assess the accuracy of the model. Later, Molecular graphics visualization program PyMoL and Chimera were used for visualization and editing of PDB models.

## Additional files

Additional file 1:
**Cloning and schematic representation of PschSOD cloning.**
Additional file 2:
**Optimization of induction temperature for PschSOD recombinant expression.**
Additional file 3:
**Effect of Copper (CuSO**_**4**_ **) and Zinc (ZnSO**_**4**_ **) supplementation on recombinant BL21 (DE3) viability.**
Additional file 4:
**Effect of Copper (CuSO**_**4**_ **) and Zinc (ZnSO**_**4**_ **) supplementation on PschSOD expression and activity.**
Additional file 5:
**Effect of denaturating and proteolytic agents on PschSOD O**_**2**_^.-^ **dismutase activity (** ***in gel*** **densitometry analysis of SOD bands).**

## Abbreviations

CD: Circular Dichroism
DCFH-DA: 2′,7′-dichlorfluorescein-diacetate
DDC: Diethyldithiocarbamate
DTPA: Diethylenetriamine pentaacetic acid
*E.coli*: *Escherichia coli*
H_2_O_2_: Hydrogen peroxide
IPTG: Isopropyl *β*-D-1-thiogalactopyranoside
PschSOD: *Pisum sativum* SOD II
KCN: Potassium cyanide
NaN_3_: Sodium azide
SOD: Superoxide dismutase
