# Supplementary material for: Heterologous expression and biochemical characterization of a highly active and stable chloroplastic CuZn-superoxide dismutase from Pisum sativum
Source: BMC Biotechnol. 2015 Feb 8;15(1):3. doi: 10.1186/s12896-015-0117-0 (PMC4333176; doi:10.1186/s12896-015-0117-0)
Supplement: Additional file 1: — Cloning and schematic representation of PschSOD cloning. [file 12896_2015_117_MOESM1_ESM.doc]

**Additional file 1: Cloning and schematic representation of PschSOD cloning.**


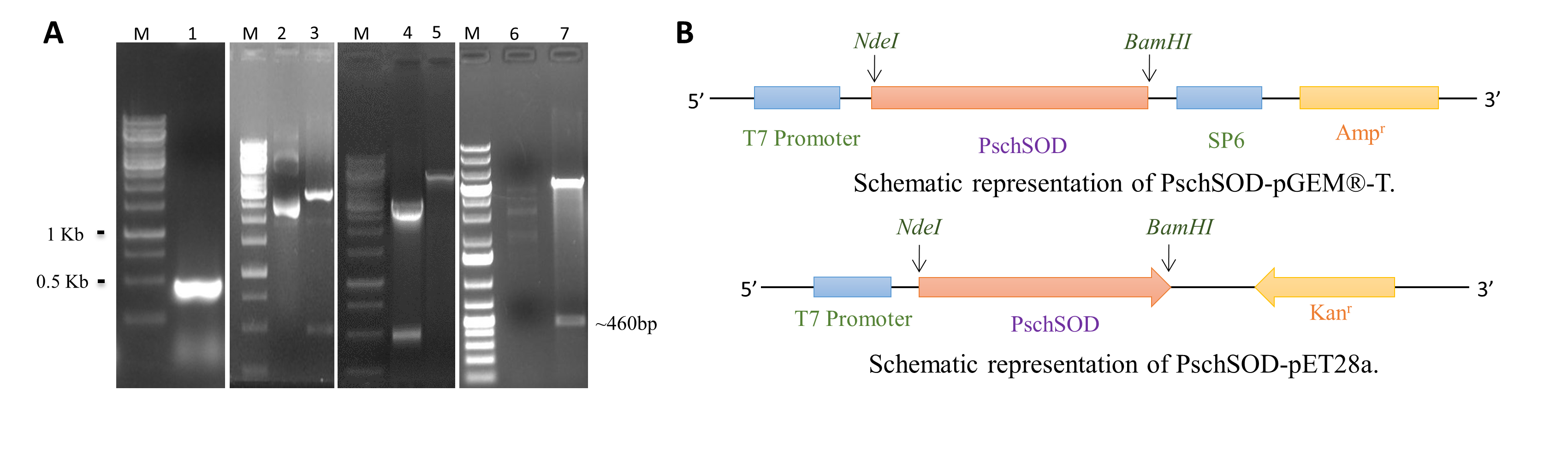


**Additional file 1: Cloning and schematic representation of PschSOD cloning.** Figure (A) represents cloning of SOD from *Pisum sativum* cDNA library to pET-28a vector. M – DNA Marker, 1- PCR with gene specific primers using cDNA library as template, 2- Uncut plasmid of SOD-pGEM-T, 3- restriction digestion of SOD-pGEM-T with NdeI and BamHI, 4- restriction digestion of SOD-pGEM-T with NdeI and BamHI 5- restriction digestion of pET-28a with *NdeI* and *BamHI*, 6- Uncut plasmid of SOD-pET-28a, 7- restriction digestion of SOD-pET-28a with *NdeI* and *BamHI*. Figure (B) represents the schematic diagrams of the both clones.
