# Supplementary material for: Heterologous expression and biochemical characterization of a highly active and stable chloroplastic CuZn-superoxide dismutase from Pisum sativum
Source: BMC Biotechnol. 2015 Feb 8;15(1):3. doi: 10.1186/s12896-015-0117-0 (PMC4333176; doi:10.1186/s12896-015-0117-0)
Supplement: Additional file 2: — Optimization of induction temperature for PschSOD recombinant expression. [file 12896_2015_117_MOESM2_ESM.doc]

**Additional file 2: Optimization of induction temperature for PschSOD recombinant expression.**


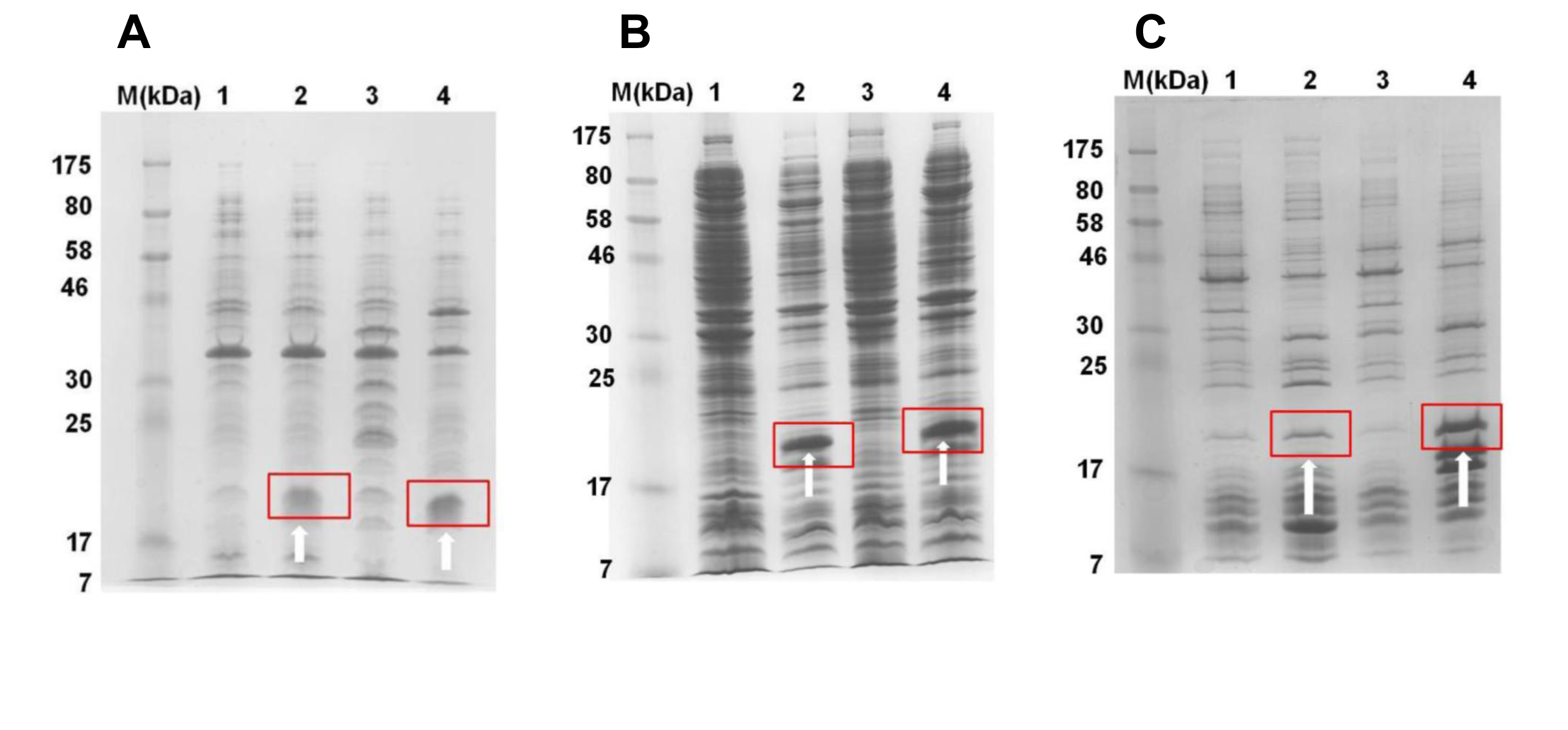


**Additional file 2: Optimization of induction temperature for PschSOD recombinant expression.** 12% SDS-PAGE analysis of (A) Periplasmic fraction (B) Cytoplasmic fraction (C) Inclusion body is depicted. In all three fractions, bacterial cells post 5-h induction period at 37 ^o^C is shown in panel 1 and 2 respectively under non-induced and induced conditions respectively; while18 ^o^C induction temperature has been shown in panel 3 and 4 under non-induced and induced conditions. Standard molecular marker for the denaturating PAGE has been marked as ‘M’. Red box with solid arrow mark the His-tagged fusion protein in the induced fractions. In periplasmic fraction 15μg of protein was used while for cytoplasmic and inclusion body fraction 25μg of protein was used for analysis.
