# Supplementary material for: Heterologous expression and biochemical characterization of a highly active and stable chloroplastic CuZn-superoxide dismutase from Pisum sativum
Source: BMC Biotechnol. 2015 Feb 8;15(1):3. doi: 10.1186/s12896-015-0117-0 (PMC4333176; doi:10.1186/s12896-015-0117-0)
Supplement: Additional file 3: — Effect of Copper (CuSO 4 ) and Zinc (ZnSO 4 ) supplementation on recombinant BL21 (DE3) viability. [file 12896_2015_117_MOESM3_ESM.doc]

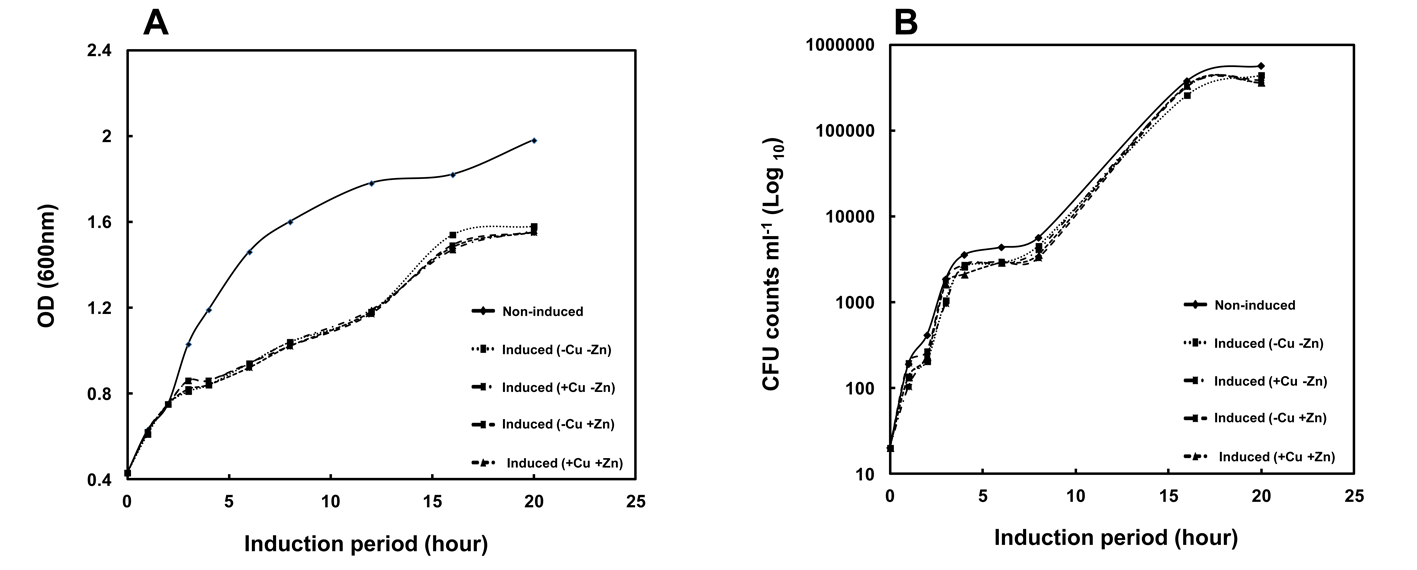
**Additional file 3:** **Effect of Copper (CuSO_4_) and Zinc (ZnSO_4_) supplementation on recombinant BL21 (DE3) viability.**

**Additional file 3: Effect of Copper (CuSO_4_) and Zinc (ZnSO_4_) supplementation on recombinant BL21 (DE3) viability.** (A) Induction period bacterial growth curve at 18^ο^C at 250μM each of CuSO_4_ and ZnSO_4_ is depicted as (A) OD600 nm (B) Colony Forming Unit (CFU) count per ml. Details of supplementation condition of Cu and Zn is mentioned in the figure.
