# Supplementary material for: Heterologous expression and biochemical characterization of a highly active and stable chloroplastic CuZn-superoxide dismutase from Pisum sativum
Source: BMC Biotechnol. 2015 Feb 8;15(1):3. doi: 10.1186/s12896-015-0117-0 (PMC4333176; doi:10.1186/s12896-015-0117-0)
Supplement: Additional file 4: — Effect of Copper (CuSO 4 ) and Zinc (ZnSO 4 ) supplementation on PschSOD expression and activity. [file 12896_2015_117_MOESM4_ESM.doc]

**Additional file 4:** **Effect of Copper (CuSO_4_) and Zinc (ZnSO_4_) supplementation on PschSOD expression and activity.**

**
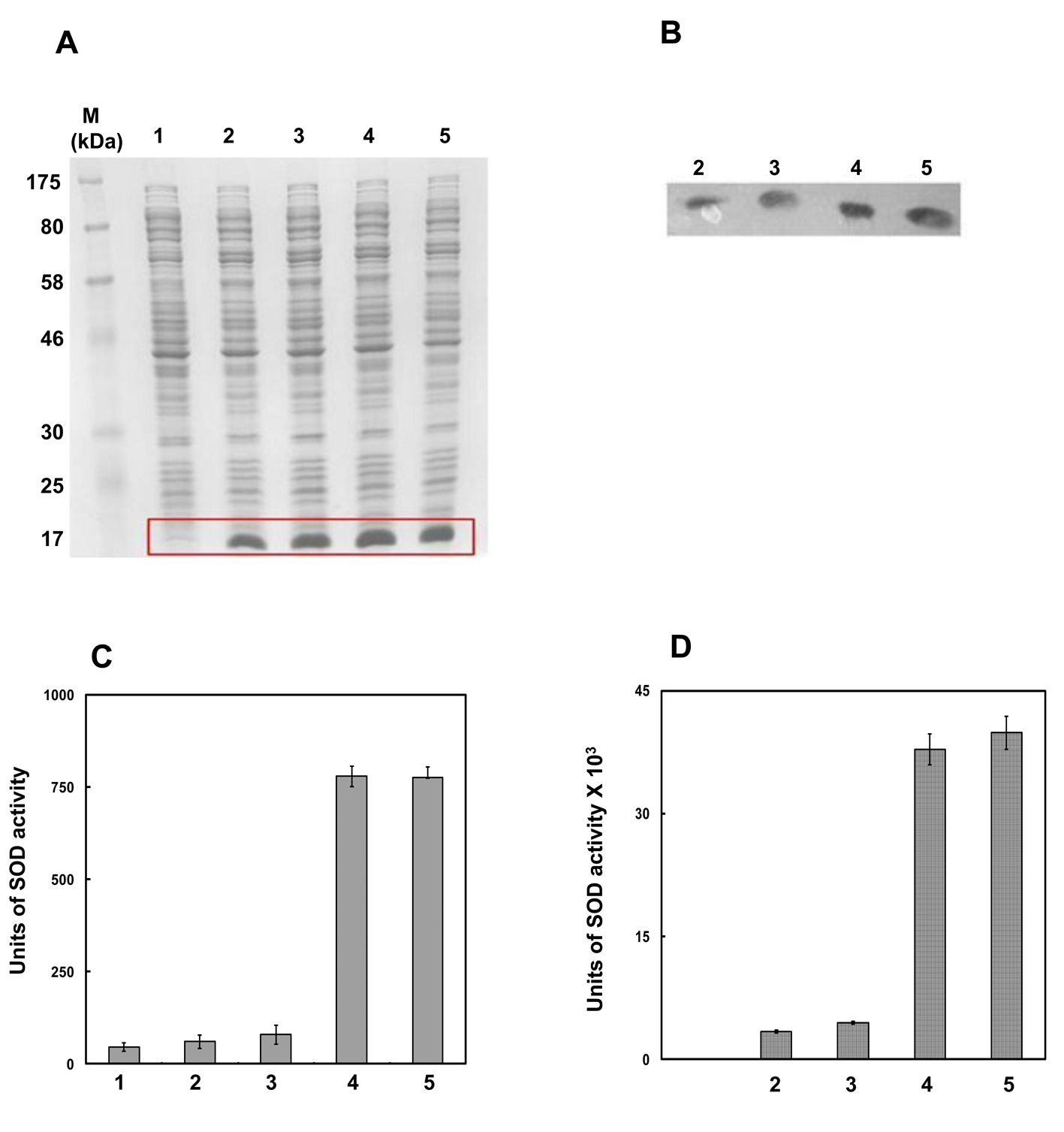
**

**Additional file 4:** **Effect of Copper (CuSO_4_) and Zinc (ZnSO_4_) supplementation on PschSOD expression and activity.** Effect of copper and zinc addition during induction period on PschSOD expression is shown as 12% SDS-PAGE in (A) while western blot using anti-his antibody is shown in (B). 25μg for SDS-PAGE and 5μg of protein was used for western blot analysis. (C) and (D) show SOD activity at below mentioned conditions. SOD activity per mg of protein is represented. In all the figures, Panel 1 represent non-induced fraction, 2-induced fraction with no Cu or Zn, 3- induced fraction with Zn but no Cu, 4- induced fraction with Cu but no Zn, 5- induced fraction with both Cu and Zn.
