# Supplementary material for: Heterologous expression and biochemical characterization of a highly active and stable chloroplastic CuZn-superoxide dismutase from Pisum sativum
Source: BMC Biotechnol. 2015 Feb 8;15(1):3. doi: 10.1186/s12896-015-0117-0 (PMC4333176; doi:10.1186/s12896-015-0117-0)
Supplement: Additional file 5: — Effect of denaturating and proteolytic agents on PschSOD O 2 .- dismutase activity ( in gel densitometry analysis of SOD bands). [file 12896_2015_117_MOESM5_ESM.doc]

**Additional file 5: Effect of denaturating and proteolytic agents on PschSOD O_2_^∙-^****dismutase activity (*in gel* densitometry analysis of SOD bands)**

**
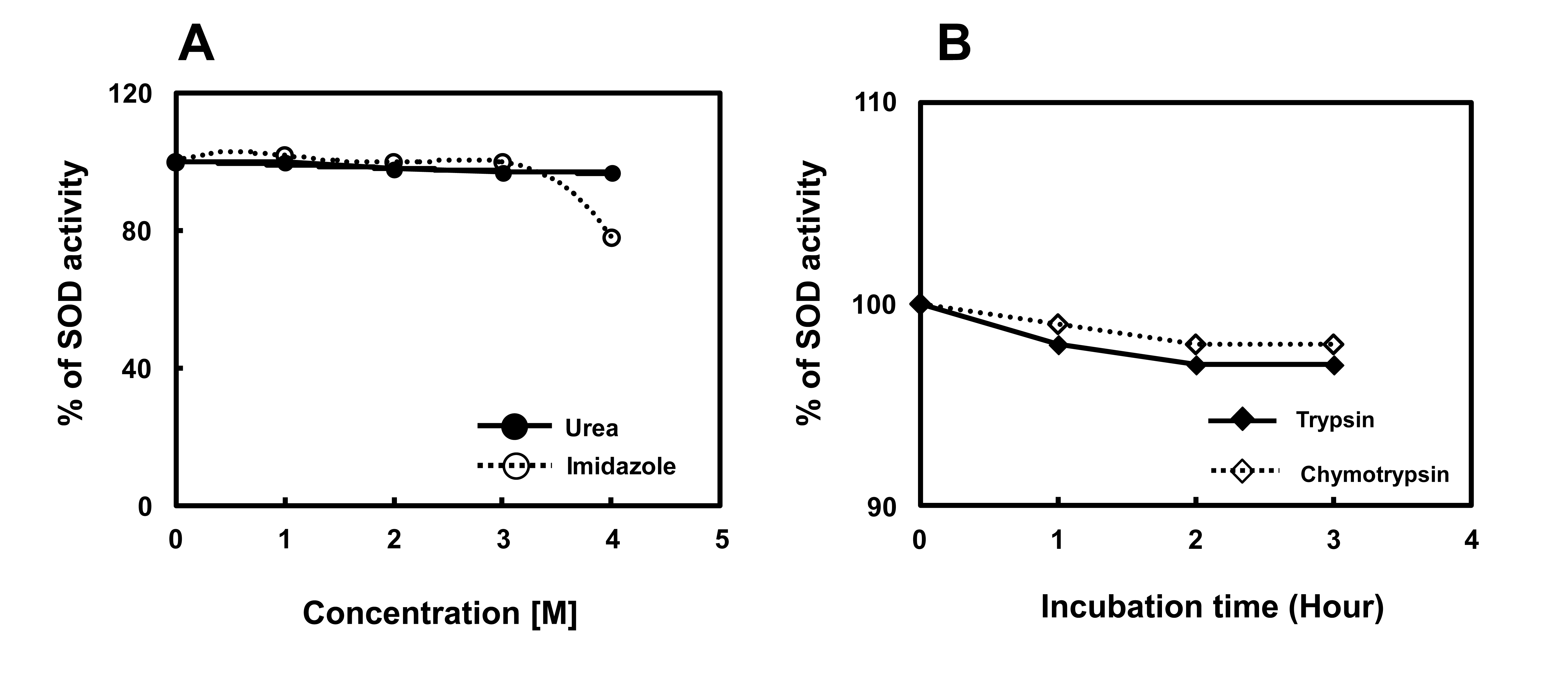
**

**Additional file 5: Effect of denaturating and proteolytic agents on PschSOD O_2_^∙-^****dismutase activity (*in gel* densitometry analysis of SOD bands).** The *in gel* densitometry analysis of SOD bands were done with the help of Quantity One software (Biorad). For denaturating agents; Additional file 3A, 0M concentration of Urea and Imidazole were serve as control corresponding to 100% activity and for Trypsin and Chymotrypsin initial activity (at 0 hour) refers to the control and corresponds to its 100% activity.
